# Pre-emptive increase in the filgrastim dose based on the CD34^+^ cell count on day four of autologous stem cell mobilization

**DOI:** 10.1016/j.htct.2024.05.016

**Published:** 2024-09-23

**Authors:** Paulla Rayane Chaves Utsch, Fernando Barroso Duarte, Abrahão Elias Hallack Neto

**Affiliations:** a Universidade Federal de Juiz de Fora (UFJF), Juiz de Fora, MG, Brazil; b Universidade Federal do Ceará (UFC), Fortaleza, CE, Brazil

Mobilization is a fundamental step in autologous hematopoietic stem cell transplantation, making hematopoietic progenitor stem cells (HPSCs), identified by the CD34 surface marker (CD34^+^), available in the peripheral blood (PB) for collection by apheresis.^1^ While filgrastim is the safest and least costly mobilization agent, it yields insufficient HPC to make transplantation possible in nearly 30 % of patients.^2^

The enumeration of CD34^+^ cells in PB is a broadly adopted predictor of successful HPSC collection³. A CCXR4 inhibitor (plerifaxor) reduces the risk of collection failure and can be helpful in rescuing poor mobilization; however, its high cost makes regular use in developing countries unfeasible.^4^^,^^5^

We developed a retrospective study to evaluate the effectiveness of a pre-emptive increase in filgrastim dose based on CD34^+^ enumeration on the fourth day of filgrastim administration and to identify factors influencing mobilization success.

Patients with multiple myeloma (MM) and lymphoma eligible for hematopoietic stem cell transplantation were retrospectively evaluated from a sample of the University Hospital of the Federal University of Juiz de Fora. The calculated sample size was 114 patients in order to achieve a significance level of less than 5 % (*p*-value < 0.05) and a 95 % confidence interval. We employed the Pearson test for statistical analysis of non-parametric data and the *t*-test for parametric data. The Poisson regression model was employed in the multivariate analysis.

This study was approved by the Research Ethics Committee of the Federal University of Juiz de Fora (# CAE 71613921.0.0000.5147).

From the first to the fourth day, all patients received approximately 10 mcg/kg/day of filgrastim, with a mean dose of 11.1 mcg/kg/day. On the morning of the 4th day, if the CD34^+^ cell count was ≥10 cells/µL, the patient was started on stem cell collection via apheresis; otherwise, the dose was increased to 20 mcg/kg/day. Then, on the 5th day, a new cytometric analysis was performed to determine the CD34^+^ cell count with harvesting beginning if there were ≥9 cells/µL.

Gender, age group, underlying disease, number of previous therapies, therapeutic response, and prior radiotherapy were assessed to identify any impact on mobilization.

Between 2018 and 2023, 116 patients were evaluated; the ages ranged between 16 and 75 years, with a median of 32 years for patients with lymphoma and 58 years for those with MM (Table 1).

**Table 1:** Characteristics of the sample.

Characteristics	Mobilization		No mobilization	
Day 4	n	%	n	%
Age	49		52	
Myeloma	53	45.7	27	23.3
Male	49	42.2	11	9.5
Albumin (g/dL)	4.16		3.97	
Leukocyte count (x 10^3^ /mm³)	40,000		33,000	
Platelet count (x10^9^ /L)	230		178	
Mean dose G-CSF (mcg/kg/d)	11.94		19.98	
Number of patients lines 2 or less	73	62.9	23	19.9
Deauville response 3/VGPR	54	46.5	17	14.7
Deauville response 4–5/PR	33	28.4	10	8.6
Radiotherapy Yes	12	10.3	6	5.2
Total: 116	89	76.7	27	23.3
**Day 5**	**Mobilization**		**No mobilization**	
	**n**	**%**	**n**	**%**
Age	51.4		54.4	
Myeloma	12	44. 4	7	25.9
Male	49	42.2	11	9.5
Albumin (g/dL)	3.99		3.93	
Leukocyte count (x 10^3^ /mm³)	35,000		28,000	
Platelet count (x10^9^ /L)	186		162	
Mean dose G-CSF (mcg/Kg/d)	20		19.98	
Number of patients lines 2 or less	14	51.9	9	33.3
Deauville response 3/VGPR	11	40.8	6	22.2
Deauville response 4–5/PR	6	22.2	4	14.8
Radiotherapy Yes	6	22.2	0	0
Total: 27	18	66.7	9	33.3

VGPR: very good partial response; PR: partial response.

Albumin, leukocytes, and platelets are depicted by their mean values.

The success rate, on Day 4 was 76.7 % (*n* = 89). According to the literature, the chance of unsuccessful mobilization with the traditional dose (10 mcg/kg/day) is approximately 30 %, very similar to that found in our study, in which we had a 24 % failure rate.^3^^,^^6^ For patients with MM, increasing the dose on the 4th day of mobilization based on the CD34^+^ cell count led to a success rate of 68 %, which is higher than the 48 % described by Sinha et al.^7^ The same author reported a CD34^+^ cell count ≥10 cells/µL in only 37 % of patients with lymphoma on the 5th day with a dose of 10 mcg/kg/day versus the 62.5 % found in the current study.^7^ Another investigation demonstrates that remobilization proves ineffectual in 81.6 % when solely employing G-CSF and 73.5 % with chemomobilization. These elevated rates of failure starkly contrast with our research findings, underscoring the imperative nature of timely interventions to ensure the efficacious harvest of CD34^+^ cells.^8^

In the subgroup of patients with dose adjustment, we observed that, in the parametric evaluation, albumin levels emerged as a statistically significant predictor (p-value = 0.04). However, this outcome did not persist in the multivariate analysis. The CD34^+^ cell count proved to be the only reliable predictor of successful mobilization, since none of the other variables investigated showed a statistically significant relationship capable of predicting the outcome of the process. This emphasizes the critical importance of conducting interventions guided by the CD34^+^ cell count.

Moreover, we found that out of the 27 patients requiring dose adjustment, only three did not meet the 9 cells/kg cutoff. Subsequently, after a five-day mobilization period, an additional six patients failed to achieve the desired count, necessitating referral for chemomobilization.

In Brazil, it is not possible to use plerixafor in the public health system. In view of the findings of the present study, we believe that a pre-emptive increase in the dose of filgrastim based on the CD34^+^ cell count on the fourth day can increase the chance of achieving the appropriate number of cells, avoiding the toxicity of chemomobilization and the costs of plerixafor. These encouraging results are motivation for us to conduct a prospective randomized study to evaluate the real benefit of this strategy in the future, especially in patients with MM.

## Conflicts of interest

The authors declare no conflicts of interest
